# Estimation of phase distortions of the photoplethysmographic signal in digital IIR filtering

**DOI:** 10.1038/s41598-024-57297-3

**Published:** 2024-03-19

**Authors:** Denis G. Lapitan, Dmitry A. Rogatkin, Elizaveta A. Molchanova, Andrey P. Tarasov

**Affiliations:** 1 Moscow Regional Research and Clinical Institute (“MONIKI”), 129110 Moscow, Russia; 2 National Research Centre “Kurchatov Institute”, 123182 Moscow, Russia

**Keywords:** Biomedical engineering, Electrical and electronic engineering, Optoelectronic devices and components

## Abstract

Pre-processing of the photoplethysmography (PPG) signal plays an important role in the analysis of the pulse wave signal. The task of pre-processing is to remove noise from the PPG signal, as well as to transmit the signal without any distortions for further analysis. The integrity of the pulse waveform is essential since many cardiovascular parameters are calculated from it using morphological analysis. Digital filters with infinite impulse response (IIR) are widely used in the processing of PPG signals. However, such filters tend to change the pulse waveform. The aim of this work is to quantify the PPG signal distortions that occur during IIR filtering in order to select a most suitable filter and its parameters. To do this, we collected raw finger PPG signals from 20 healthy volunteers and processed them by 5 main digital IIR filters (Butterworth, Bessel, Elliptic, Chebyshev type I and type II) with varying parameters. The upper cutoff frequency varied from 2 to 10 Hz and the filter order—from 2nd to 6th. To assess distortions of the pulse waveform, we used the following indices: skewness signal quality index (*S*_*SQI*_), reflection index (*RI*) and ejection time compensated (*ETc*). It was found that a decrease in the upper cutoff frequency leads to damping of the dicrotic notch and a phase shift of the pulse wave signal. The minimal distortions of a PPG signal are observed when using Butterworth, Bessel and Elliptic filters of the 2nd order. Therefore, we can recommend these filters for use in applications aimed at morphological analysis of finger PPG waveforms of healthy subjects.

## Introduction

Photoplethysmography (PPG) is a simple optical technique for detection of blood volume changes in the microvascular bed of a biological tissue^1^. Currently, PPG is actively used to assess different parameters of the cardiovascular system. The method is based on illumination of a tissue with incoherent optical radiation in the visible or near-infrared range and recording a signal that has passed through or backscattered from the tissue^2^. The signal registered in PPG consists of a variable component (AC), which is formed due to blood pulsations, and a slow varying component (DC) related to the average blood volume in a tissue^1^. Clinical applications of PPG include monitoring of pulse rate and its variability, cardiac output and respiration, assessment of microvascular blood flow and tissue viability, endothelial and vasomotor function, thermoregulation, stress, detection of atrial fibrillation, sleep disorders, etc.^1,3–7^. However, morphological analysis of the pulse waveform extracted from the AC component of a PPG signal provides other important information about the state of blood vessels, for example, their stiffness, tone, compliance, etc.^8,9^. Therefore, it is very important to use correct signal processing, as incorrect filtering can distort the shape of the pulse wave^10^. This is especially important when using the analysis of the second derivative of the photoplethysmogram^11^, as well as when measuring the pulse wave velocity between two points on the body using PPG technology^12–16^.

As a rule, in most modern PPG devices and systems, digital filtering is used to remove noise from signals. Noise reduction is an important step in processing PPG signals and makes it possible to extract useful information from them^17^. Depending on the tasks, researchers use different algorithms for processing the PPG signal. Some use continuous real-time signal processing, others focus on removing motion artifacts from the photoplethysmogram signal^18–20^ and still others are developing different types of filters^21–23^. Digital filters with infinite impulse response (IIR) are actively used in the PPG signal processing^17^. Unlike finite impulse response (FIR) filters, they have a simpler software implementation and allow designing classic analog filters such as Butterworth, Bessel, Chebyshev, etc.^24^. One of the properties of filtering is the time delay it introduces into the output signal^17^. FIR filters introduce longer delays since they must be of a higher order to achieve similar responses^17^. However, IIR filters are less stable and have a non-linear phase response^24,25^. Therefore, the effect of IIR filters on the PPG waveform requires a more detailed study.

One of the most used IIR filters in PPG signal processing is the Butterworth filter, since it has a flat amplitude response in the passband, which guarantees the least signal distortion^23,24^. For this reason, the Butterworth filter has found wide application in processing PPG signals^26,27^. At the same time, researchers also use other IIR filters. For example, S.M. Lopez-Silva et al. used a low-pass and a band-pass Bessel filter to obtain DC and AC components of the PPG signal, respectively, for oxygen saturation measurements^28^. Many works are devoted to the effect of filtering on pulse rate variability^29,30^. In particular, it was found that pulse rate variability information can be reliably extracted from PPG signals filtered using lower low cutoff frequencies and elliptic IIR or equiripple or Parks–McClellan FIR filters^31^. It has recently been shown that the Chebyshev II filter of the 4th order is optimal in terms of the PPG signal quality^23^. Liang et al. used the 0.5–10 Hz Chebyshev II band-pass filter to remove the noise from PPG signals^32^. However, this filter has not been investigated for the presence of phase distortions of the pulse waveform. There is no clear understanding of which of the IIR filters is most suitable for PPG signals from the point of view of the least signal distortions.

As known, the pulse wave consists of direct systolic and reflected diastolic waves, as well as a dicrotic notch, which characterizes the moment of aortic valve closure^10,33^. Herewith, the shape of the pulse wave in PPG depends on many factors such as the measurement location on the body, the elasticity of vessel walls, etc.^34–36^. The frequency range of the PPG signal is approximately from 0.01 to 10 Hz^2^. Herewith, the strongest oscillations caused by heart contractions are observed in the region of 1 Hz. Thus, the lower limit of the filter's bandwidth should be somewhere between 0.01 and 1 Hz. J. Allen and A. Murray investigated the effect of lower cutoff frequency of the filtering on the pulse waveform and found that when the lower cutoff is increased above 0.2 Hz, distortion of the direct and reflected waves is observed^37^. With a decrease in the upper cutoff frequency of the band-pass filtering, the dicrotic notch of the pulse wave is damped^38^. It was shown that this does not reduce the accuracy of the oxygen level measurements in pulse oximetry^38^. H. Liu et al. investigated the effect of PPG signal morphology on the filtering-induced time shift and found that measurement site and type of pulse feature can significantly influence the timing of feature point on filtered PPG signals^39^. However, these studies provide fragmentary data on the influence of filtering parameters on the shape of the PPG signal. We did not find more systematic and detailed studies of the effect of filtering parameters (bandwidth, filter order, etc.) on the pulse waveform. Thus, the parameters of the PPG signal filtering for more accurate reproduction of the pulse wave shape are unknown.

The aim of this work is to study the influence of the band-pass IIR filtering parameters (lower and upper cutoff frequencies, type and order of a filter) on the PPG waveform as applied to the task of its morphological analysis. In this work, we investigated 5 types of IIR filters: Butterworth, Bessel, Elliptic, Chebyshev I and II. Our hypothesis is that the Butterworth filter provides minimal distortions of the PPG signal and that increasing the filter order and narrowing the bandwidth will lead to significant changes in the pulse waveform.

## Theoretical background

First, let us review the theoretical basis of band-pass filtering. As an example, consider a second order band-pass filter that can be implemented as a consistent connection of a low-pass filter and a high-pass filter. The transfer function of such filter can be represented as^40^:

1 $$H\left(s\right)=\frac{\frac{{\omega }_{0}}{Q}s}{{s}^{2}+\frac{{\omega }_{0}}{Q}s+{\omega }_{0}^{2}},$$

where *s* = *jω*, *ω*_*0*_ is the central frequency of the filter passband, *Q* is the quality factor of the filter. The quality factor is defined as:

2 $$Q=\frac{{f}_{0}}{\Delta f},$$

where *Δf* is the bandwidth of the filter. For a band-pass filter *Δf* = *f*_*H*_ – *f*_*L*_, where *f*_*L*_ and *f*_*H*_ are the lower and upper cutoff frequencies of the filter, respectively.

The phase response of a band-pass filter is:

3 $$\phi \left(\omega \right)=arg\left\{H(s)\right\}=\frac{\pi }{2}-{\text{arctan}}\left(\frac{2Q\omega }{{\omega }_{0}}+\sqrt{4{Q}^{2}-1}\right)-{\text{arctan}}\left(\frac{2Q\omega }{{\omega }_{0}}-\sqrt{4{Q}^{2}-1}\right),$$

The group delay of a filter is defined as follows^24^:

4 $${\tau }_{g}\left(\omega \right)=-\frac{d\phi \left(\omega \right)}{d\omega }.$$

Substituting (3) in (4), we obtain:

5 $${\tau }_{g}\left(\omega \right)=\frac{2Q}{{\omega }_{0}}\left(\frac{1}{1+{\left(\frac{2Q\omega }{{\omega }_{0}}+\sqrt{4{Q}^{2}-1}\right)}^{2}}+\frac{1}{1+{\left(\frac{2Q\omega }{{\omega }_{0}}-\sqrt{4{Q}^{2}-1}\right)}^{2}}\right).$$

**Figure 1:**
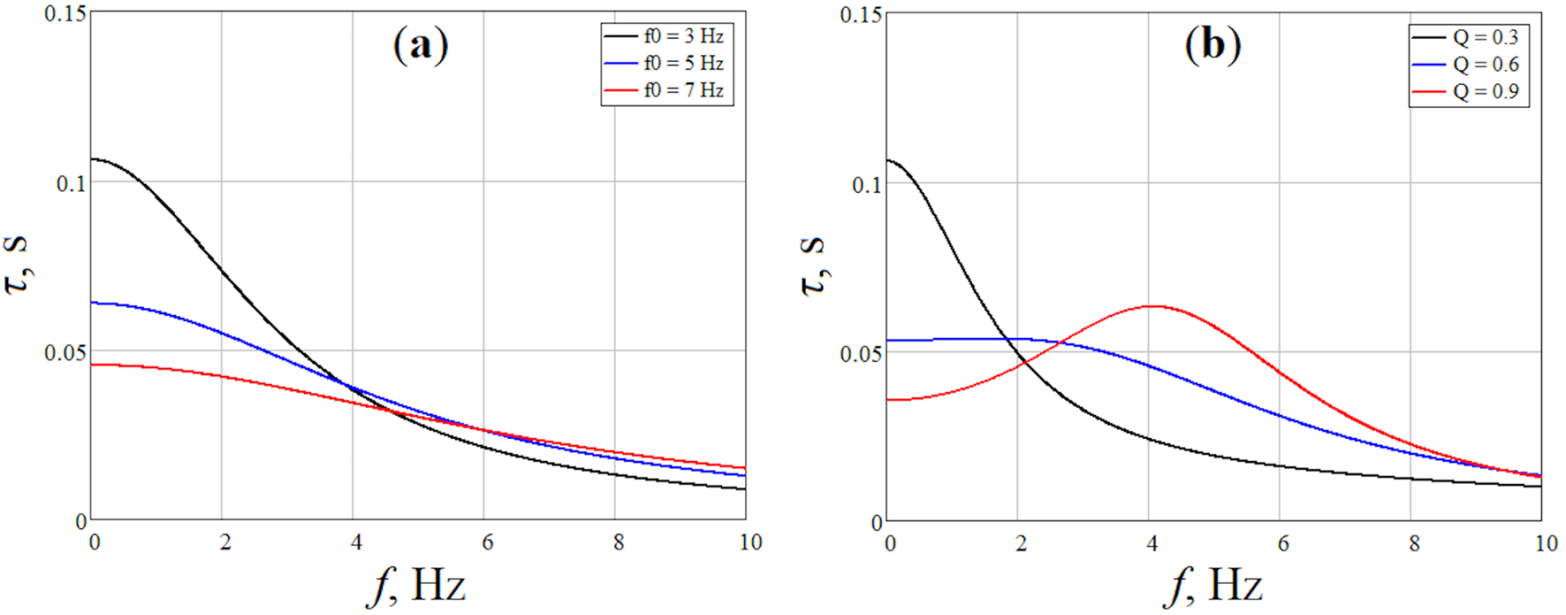
Simulated dependence of the group delay of a 2nd order band-pass filter on frequency for different values of central frequency *f*_*0*_ (**a**) and quality factor *Q* (**b**).

Thus, the group delay of the filter depends on the central frequency of the passband and the quality factor. Let us make a theoretical calculation of *τ*_*g*_(*ω*) for different values of *ω*_*0*_ and *Q*. We modeled the dependence *τ*_*g*_(*ω*) for different central frequency at a fixed quality factor (*Q* = 0.5) and different *Q* at a fixed frequency (*f*_*0*_ = 5 Hz). The results are presented in Fig. 1. As can be seen, the group delay can reach values on the order of 0.1 s for low frequencies. Since the group delay of the filter depends non-linearly on the frequency, each harmonic of the PPG signal will be processed with its own delay. Together, this will lead to a distortion of the pulse waveform and a change in its amplitude and time characteristics.

Based on theoretical data, we can conclude that the phase distortions of the PPG signal depend mainly on the central frequency of the passband and the quality factor, which are determined by the bandwidth, type and order of a filter. Thus, in order to systematically study the change in the pulse wave shape during the filtering process, it is necessary to record a raw PPG signal from biological tissue, pass it through a band-pass filter with varying parameters and compare it with the original unfiltered signal. The detailed research methodology is presented in the “Materials and methods” section.

## Results

**Figure 2:**
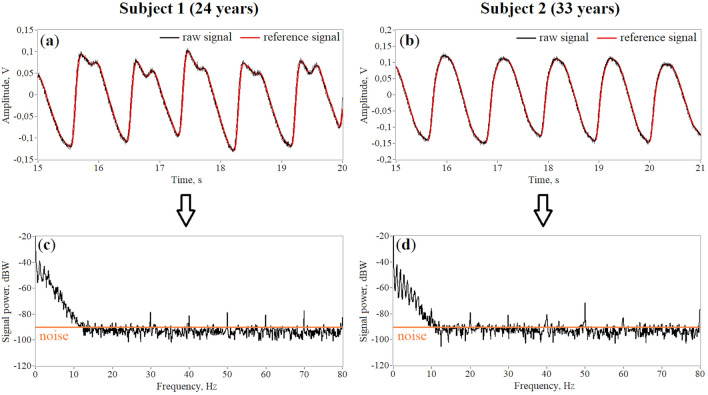
Examples of recorded raw PPG signals of 2 types: with a pronounced diastolic wave (**a**) and without it (**b**). The reference signal is the raw signal smoothed using a moving average with a window of 3.125 ms. Power spectrum of raw signals from subject 1 (**c**) and subject 2 (**d**).

A total of 20 fingertip PPG signals were recorded. The signals were divided into 2 subgroups: 1—with a pronounced diastolic wave (*n* = 10), 2—with a barely noticeable or absent diastolic wave (*n* = 10). Examples of raw signals from two subgroups are shown in Fig. 2. The difference in the shape of the PPG signals between two subjects is associated with age. It is known that vascular stiffness increases with age, which is reflected in the pulse waveform in the form of a weakening of the diastolic wave^35^. Thus, these examples of signals reveal intra-subject variability in the pulse waveforms that were analyzed in our study. The reference signal was obtained by smoothing the raw PPG signal using a moving average. As can be seen from Fig. 2, the reference signal completely follows the contour of the raw signal. This allows us to compare the filtered signals to a reference signal rather than to a raw signal, which contains high-frequency noise. We also plotted the power spectra of presented signals to estimate the frequency range of the useful signal and noise (see Fig. 2c,d). As can be seen, the useful harmonics of both PPG signals are concentrated to approximately 10 Hz. The noise has a uniform distribution over the entire frequency band with separate harmonics at 30, 50 Hz, etc. and its level is approximately − 90 dB (orange line in Fig. 2c,d).

**Figure 3:**
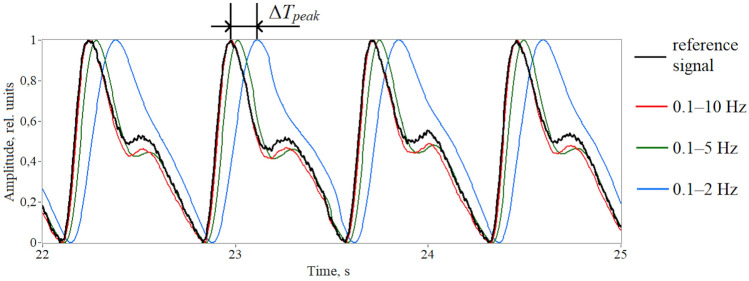
Processing of the raw PPG signal (black line) using a 2nd order Butterworth band-pass filter with a various upper boundary of the frequency range: 0.1–10 Hz (red line), 0.1–5 Hz (green line) and 0.1–2 Hz (blue line). The *HR* is 87 beats per minute (1.45 Hz).

Figure 3 shows the reference PPG signal and the signals obtained by band-pass filtering the reference signal with a 2nd order Butterworth filter with different upper cutoff frequencies (10, 5 and 2 Hz). As can be seen, the reference signal contains high-frequency electrical noise, which is suppressed by filtering. At the same time, filtering of the PPG signal leads to distortions of the pulse waveform. A decrease in the upper boundary of the filtering frequency range leads to damping of the dicrotic notch because higher frequency harmonics of the signal are filtered out. As well, narrowing the filter bandwidth leads to a decrease in the central frequency of the passband, which, in turn, leads to an increase in the group delay of the filter in accordance with (5) (see Fig. 1a). Thus, a phase shift of the pulse wave relative to the reference signal arises.

**Figure 4:**
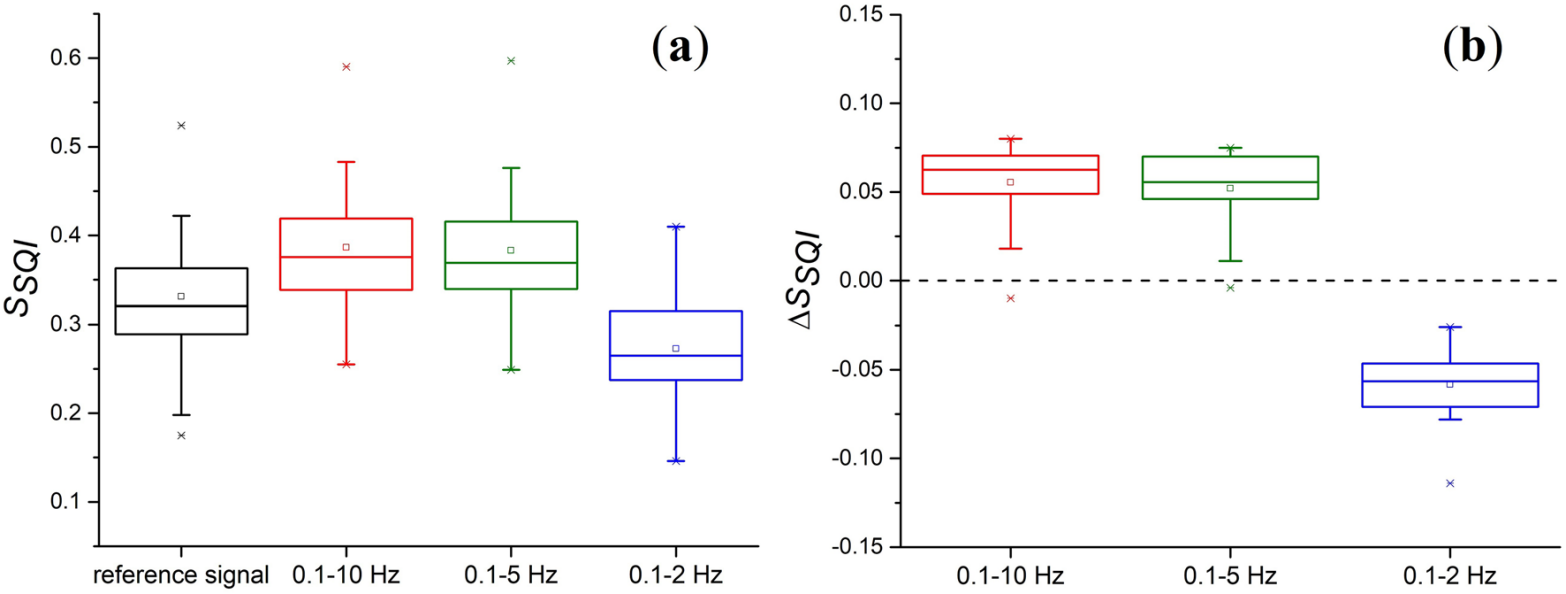
(**a**) Box diagrams of skewness signal quality index (*S*_*SQI*_) for all subjects when the PPG signal is processed by a 2nd order Butterworth filter with varying upper cutoff frequency; (**b**) Deviations of the *S*_*SQI*_ of the filtered signal from the reference one. The dotted line shows the zero level.

The calculated values of *S*_*SQI*_ for the reference signal and for signals filtered in all three frequency ranges are presented in the form of box plots in Fig. 4a. *S*_*SQI*_ was calculated for all PPG signals (*n* = 20). Filtering in the ranges of 0.1–10 Hz and 0.1–5 Hz gives approximately the same increase in the *S*_*SQI*_ by about 0.05 rel. units, while the range of 0.1–2 Hz reduces the index by approximately the same value of 0.05 rel. units. To assess the pulse waveform distortions caused by filtering, the deviations of *S*_*SQI*_ index from the reference signal were calculated as Δ*S*_*SQI*_ = *S*_*SQI,filter*_ – *S*_*SQI,ref*_, where *S*_*SQI,filter*_ is the skewness index of the filtered signal and *S*_*SQI,ref*_ is the skewness index of the reference signal. The results are shown in Fig. 4b. As seen, filtering in the ranges of 0.1–10 Hz and 0.1–5 Hz gives almost the same increment of the *S*_*SQI*_. Changes in the *S*_*SQI*_ are statistically significant for all three bandwidths (*p* < 0.001).

**Figure 5:**
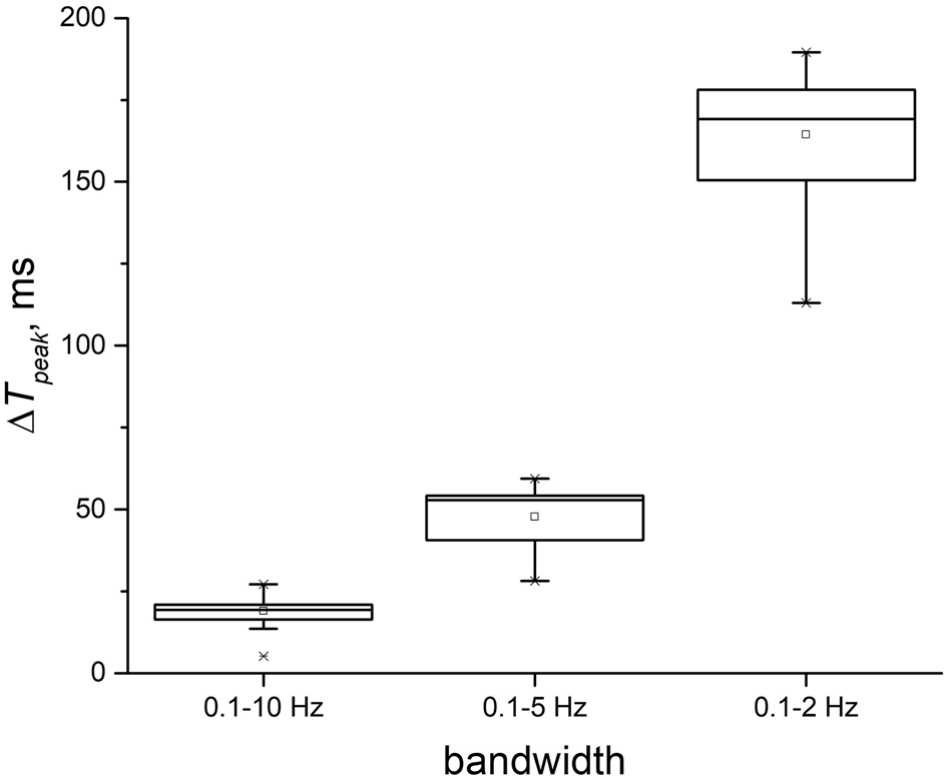
Time shift of the systolic peak during processing the PPG signal by a 2nd order Butterworth filter with varying upper cutoff frequency.

To estimate the phase shift of the PPG signal, we calculated the time deviation of the systolic peak of the filtered signal from this one of the reference signal (Δ*T*_*peak*_) for all three bandwidths. The results are presented in Fig. 5. The average value of Δ*T*_*peak*_ for all subjects was 18.7 ± 5.1 ms for a bandwidth of 0.1–10 Hz, 51.8 ± 6.8 ms for 0.1–5 Hz and 166.8 ± 13.6 ms for 0.1–2 Hz. It turns out that the time delay of the systolic peak can reach hundreds of ms.

The bandwidths of 0.1–10 Hz, 0.1–5 Hz and 0.1–2 Hz differ only in the central frequency *f*_*0*_, which is approximately 5, 2.5 and 1 Hz for them, respectively. The quality factor for all ranges is approximately the same and is about 0.5. As can be seen in Fig. 5, the phase shift of the systolic peak increases with a decrease in the central frequency of the passband that corresponds to the theoretical dependence in Fig. 1a. Thus, the minimum distortions of the pulse waveform in terms of the skewness index and time delay of the systolic peak are observed for the frequency range of 0.1–10 Hz. Therefore, further signal processing was carried out in this range.

**Figure 6:**
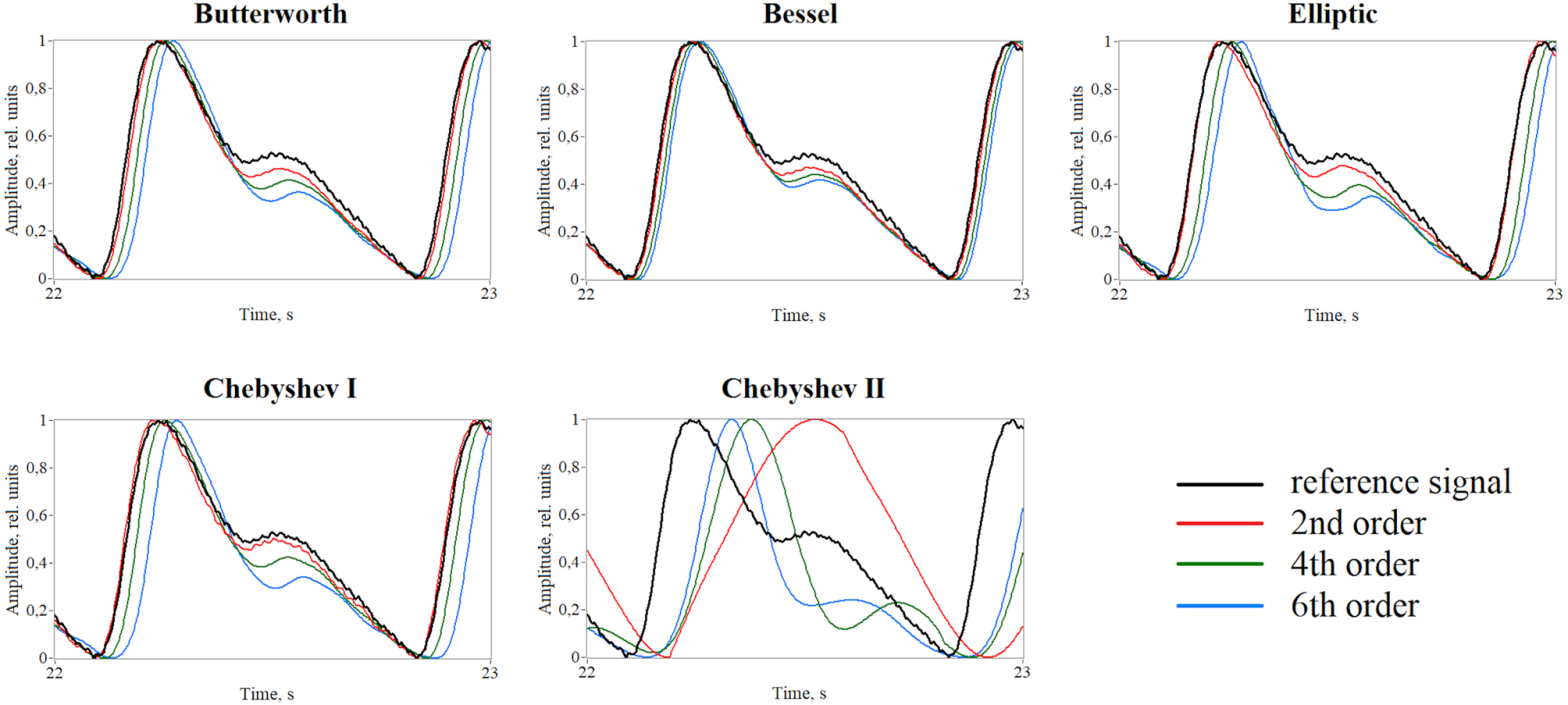
An example of processing a typical PPG waveform with different IIR filters of different orders.

Justification of the bandwidth of the PPG signal filtering allowed us to move on to the next step, namely, to study the effect of different IIR filters on the pulse waveform. Figure 6 shows pulse waveforms obtained by processing the PPG signal with Butterworth, Bessel, Elliptic, Chebyshev I and II filters of different orders (2nd, 4th and 6th). As can be seen, the Butterworth, Bessel, Elliptic and Chebyshev I filters do not strongly distort the shape of the pulse wave, while the Chebyshev II filter leads to its significant distortion. For the above filters, the following situation is observed—the higher the filter order, the smaller the amplitude of the reflected wave, while the pulse waveform practically does not change. However, for the Chebyshev II filter, the opposite situation is observed—the higher the order of the filter, the closer the PPG waveform approaches the reference signal. In addition, this filter results in a large time delay of the processed signal. This can be critical in pulse wave velocity measurements, as well as in applications where phase characteristics of the PPG signal are important^41^.

**Figure 7:**
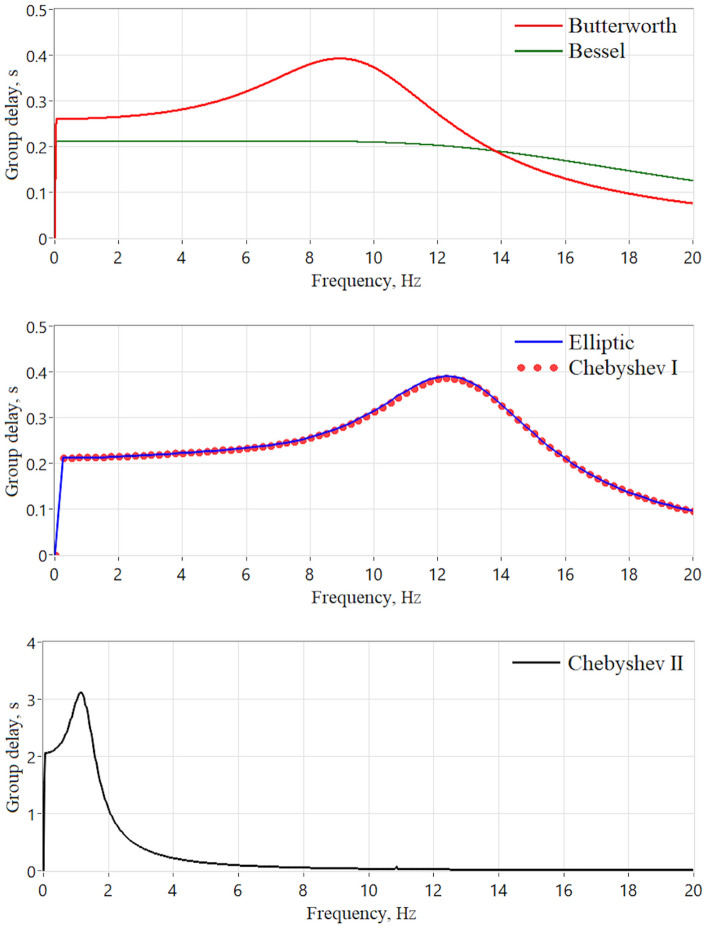
Group delay of the 0.1–10 Hz band-pass IIR filters of the 4th order, measured in LabView.

As can be seen from Fig. 6, the Bessel filter distorts the shape of the pulse wave to the least extent. As known, the Bessel filter has a maximally linear phase response in the passband, due to which the group delay of the filter is almost constant^24^. We numerically assessed the group delay of the filters in LabView using the built-in Frequency Response Function. The results are presented in Fig. 7. When performing calculations, the order of all filters was set to 4 and their bandwidth was equal to 0.1–10 Hz. The group delay is almost constant only for the Bessel filter, which explains the fact of minimal phase distortions when using it. The Chebyshev II filter has a maximally nonlinear group delay in the passband compared to other filters, so it introduces maximum distortions into the PPG signal.

**Figure 8:**
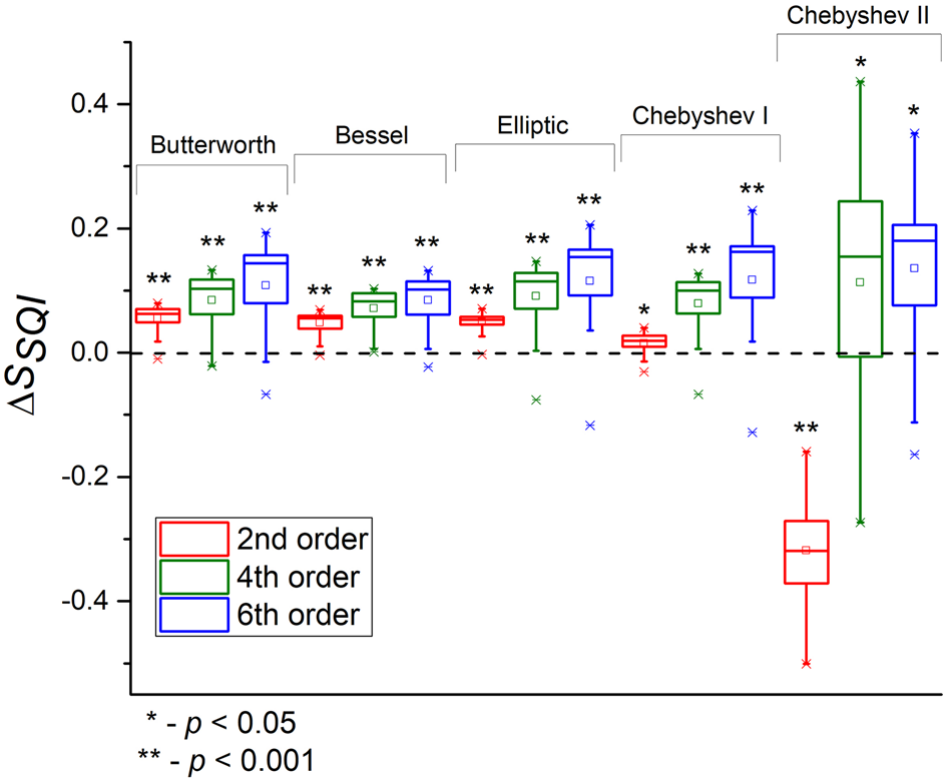
Box diagrams of skewness quality index (*S*_*SQI*_) deviations during PPG signal processing by IIR filters of different orders. The dotted line shows the zero level.

Diagrams of *S*_*SQI*_ deviations for all filters are presented in Fig. 8. As can be seen, the Butterworth, Bessel, Elliptic and Chebyshev I filters showed similar behavior. The *S*_*SQI*_ deviation grows linearly with increasing order for these filters. At the same time, changes in the *S*_*SQI*_ when using the Chebyshev II filter are very ambiguous. The 2nd filter order greatly underestimates the *S*_*SQI*_ value, and the 4th and 6th orders greatly overestimate it. Herewith, the Chebyshev II filter of the 4th order gives the largest spread of index deviations. Thus, we confirmed the result of Y. Liang et al.^23^ that the Chebyshev II filter of the 4th order can improve the PPG signal quality more effectively than other types of filters. In our case, the 6th order Chebyshev II filter also allows to greatly increase the *S*_*SQI*_ value. However, this effect is achieved due to a strong distortion of the original pulse waveform. Changes in the *S*_*SQI*_ are statistically significant for all filters: Butterworth, Bessel, Elliptic of all orders, Chebyshev I of 4th and 6th orders (*p* < 0.001) and Chebyshev I of the 2nd order, Chebyshev II of 4th and 6th orders (*p* < 0.05).

**Figure 9:**
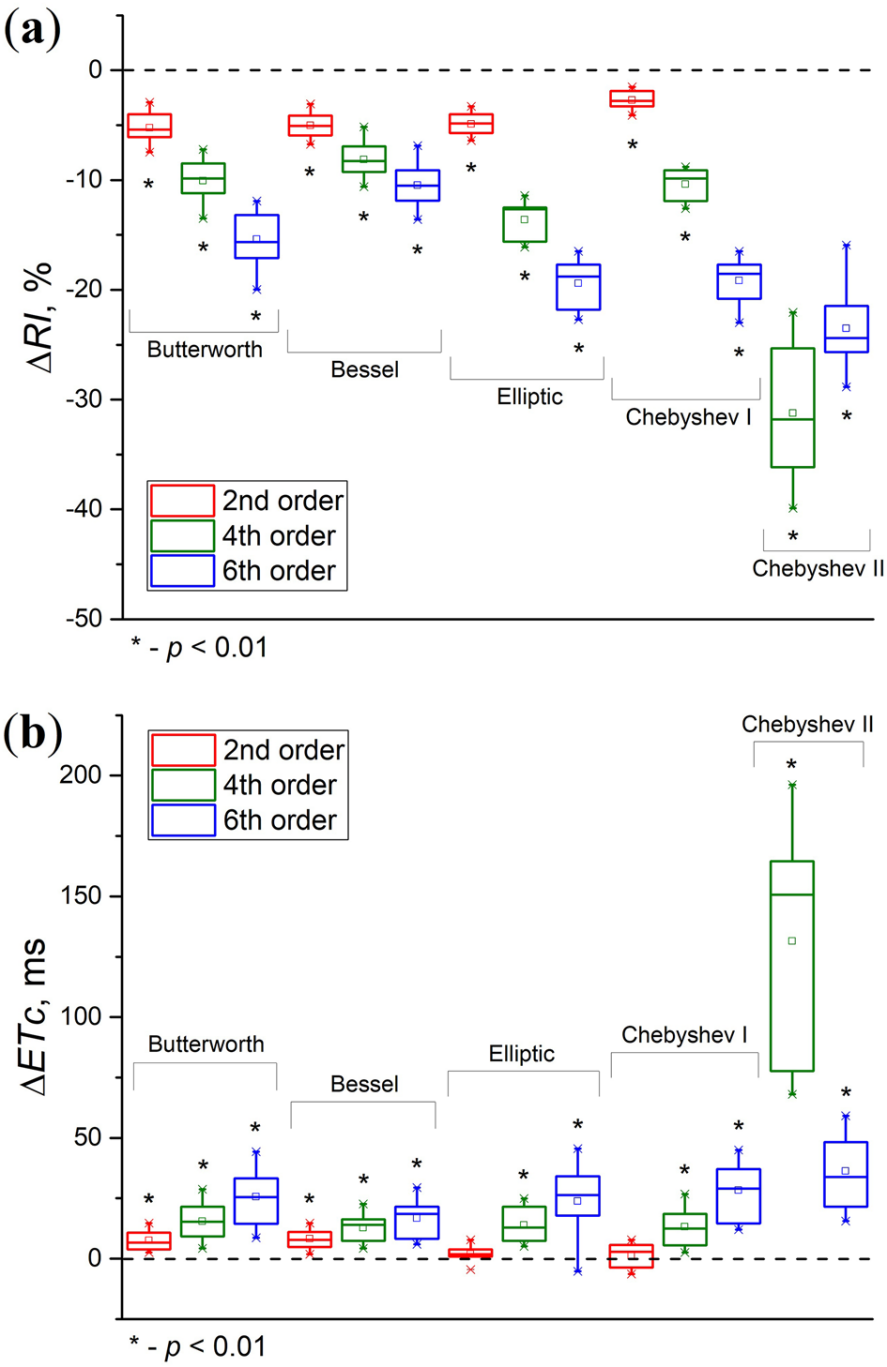
Box diagrams of deviations of the reflection index (*RI*) (**a**) and ejection time compensated (*ETc*) (**b**) during PPG signal processing by IIR filters of different orders. The dotted line shows the zero level.

Diagrams of *RI* and *ETc* deviations for PPG signals from subgroup 1 (with a pronounced dicrotic notch) when processed by all five filters are presented in Fig. 9. For the Chebyshev II filter, the results are presented only for the 4th and 6th orders, since the 2nd order does not allow the *RI* and *ETc* to be calculated due to the loss of the diastolic peak (see Fig. 6). As in the case of the *S*_*SQI*_, the Butterworth, Bessel, Elliptic and Chebyshev I filters showed quite logical and similar behavior. The *RI* falls and the *ETc* grows linearly with increasing filter order. However, this does not apply to the Chebyshev II filter. The 4th order of the Chebyshev II filter gives the maximum deviations of indices. Deviations of the *RI* are approximately 20–40%, and deviations of the *ETc* reach 100–150 ms. Changes in the *RI* are statistically significant for all filters and all orders (*p* < 0.01). Changes in the *ETc* are also statistically significant for all filters (*p* < 0.01) except the Elliptic and Chebyshev I filters of the 2nd order.

## Discussion

Thus, given the combination of all three indices (*S*_*SQI*_, *RI* and *ETc*), the minimum distortions of the pulse waveform are observed for the Chebyshev I filter of the 2nd order. However, this filter provides poor noise suppression (see Fig. 6). For this reason, we cannot recommend this filter for use. The Butterworth, Bessel and Elliptic filters of the 2nd order showed not much worse results, however, they provide good noise reduction. Therefore, these filters are more suitable for use. Thus, our initial hypothesis that the Butterworth filter causes minimal signal distortions was partially confirmed. The maximum distortions occur when using the Chebyshev II filter of the 4th order. Therefore, we cannot recommend this filter for processing the PPG signal and extracting important parameters of the cardiovascular system from the pulse waveform. It is possible that Chebyshev type I and type II filters can find application in other tasks not related to the pulse wave analysis. For example, it was recently shown that the Chebyshev II filter of 2nd, 4th and 6th orders perform the best for denoising remote PPG signals in camera-based imaging technique^42^.

It is worth noting that minimizing distortions of the PPG signal at the pre-processing stage is an important task, since errors can also arise at the post-processing stage. Thus, distortions will accumulate throughout the entire processing chain, which can lead to errors in determining the final physiological parameters. For example, when using a multi-Gaussian approach to decompose the PPG wave, the absolute error in calculating the *ETc* index reaches 15.41 ± 13.66 ms^43^. Thus, to minimize the total error in *ETc* calculation, the 2nd order Elliptic filter should be used, since it showed a statistically insignificant difference between the index value of the filtered and reference signals (see Fig. 9b). We also recommend using the Bessel filter in those applications where phase delays need to be minimized because it has the lowest and flattest group delay in the passband (see Fig. 7).

Nevertheless, our study has some limitations. Firstly, we recorded and analyzed PPG signals from the subjects' fingertips. The fingertip is the most used measurement site for recording the PPG signal. However, other locations such as the earlobe and toe are also sometimes used. Thus, PPG signals from above locations can also be additionally analyzed for completeness. Secondly, as morphological parameters of the PPG signal, we used three indices. *S*_*SQI*_ is an integral parameter in the time domain of signal and reflects the morphology of the pulse waveform. *RI* and *ETc* characterize the state of peripheral and central hemodynamics, respectively, and can be calculated only in the presence of a pronounced diastolic wave. Thus, these three indices reflect all important amplitude and temporal features of the PPG signal and, in our opinion, are sufficient to unambiguously evaluate pulse waveform distortions during filtering. However, for a more in-depth analysis of changes in the pulse waveform, you can also add other indices, for example, features of the second derivative of PPG^8^. Thirdly, we included young healthy volunteers in the study. However, despite the small age difference between the subjects, we observed noticeable differences in the PPG waveforms (see Fig. 2). Thus, our results are valid for different pulse waveforms, which are caused by different vascular stiffness, and not only for the typical PPG waveform with a pronounced diastolic wave.

The choice of a specific filter for processing PPG signals depends on the problem being solved. In this work, we investigated the influence of IIR filters on the PPG signal in relation to the task of morphological analysis of the pulse wave and selected those filters that introduce minimum signal distortions. The results obtained can be useful when choosing a specific filter in the development of PPG devices and systems. Further work will be aimed at conducting a more detailed analysis of changes in the PPG signal during filtering using other morphological indices of the pulse waveform, as well as including FIR filters in the study.

## Conclusions

In this study, we quantified the PPG waveform distortions occurring during IIR filtering. We found that reducing the upper cutoff frequency of band-pass filtering below 10 Hz leads to damping of the dicrotic notch and a phase shift of the pulse wave signal. This can be critical in applications in which the phase properties of the PPG signal play a decisive role. Therefore, an approximate bandwidth to minimize distortions of the pulse waveform is 0.1–10 Hz. We used five types of IIR filters (Butterworth, Bessel, Elliptic, Chebyshev I and II) and compared their effect on the PPG waveform with each other. The Butterworth, Bessel, Elliptic and Chebyshev I filters performed approximately the same, while the Chebyshev II filter showed completely different results. The minimal distortions of a pulse wave signal are observed when using Butterworth, Bessel and Elliptic filters of the 2nd order. Thus, these filters should be recommended for use in applications aimed at morphological analysis of finger PPG waveforms of healthy subjects.

## Materials and methods

### Instrumentation

Raw PPG signals were collected using a previously developed experimental setup, which makes it possible to record signals from a subject's fingertip in backscattering geometry^44^. The setup consists of a LED light source, a photodetector and a scheme of amplification and processing of the registered PPG signal. The setup allows to stabilize the radiation power of the LED through the use of a highly stable direct current source (QJ3003C III, Ningbo JiuYuan Electronic, China), as well as vary the source-detector distance to achieve the maximum signal-to-noise ratio. In this study, we used the green LED as a light source (L-7104GC, Kingbright, Taiwan) with a peak emission wavelength of 568 nm and the silicon photodiode (TEFD4300, Vishay, USA). The distance between the LED source and the photodiode in the experiment was 5 mm. Raw signals from skin were amplified, digitized by a 16-bit analog-to-digital converter (ADS8320EB, Texas Instruments, USA) at a sampling frequency of 320 Hz and transmitted to a computer for further processing.

### Subjects and measurement protocol

Measurements were taken on 20 healthy young volunteers (10 males and 10 females) without cardiovascular diseases. The average age of subjects is 26.2 ± 2.5 years. Only subjects with a sinus rhythm were included in the study. Signals were recorded in a sitting position. Before the experiment, the subjects rested for at least 15 min. Raw PPG signals were recorded from the tip of the left index finger for 40 s. Then the signals were stored on a computer for further processing.

### PPG signal processing

**Figure 10:**
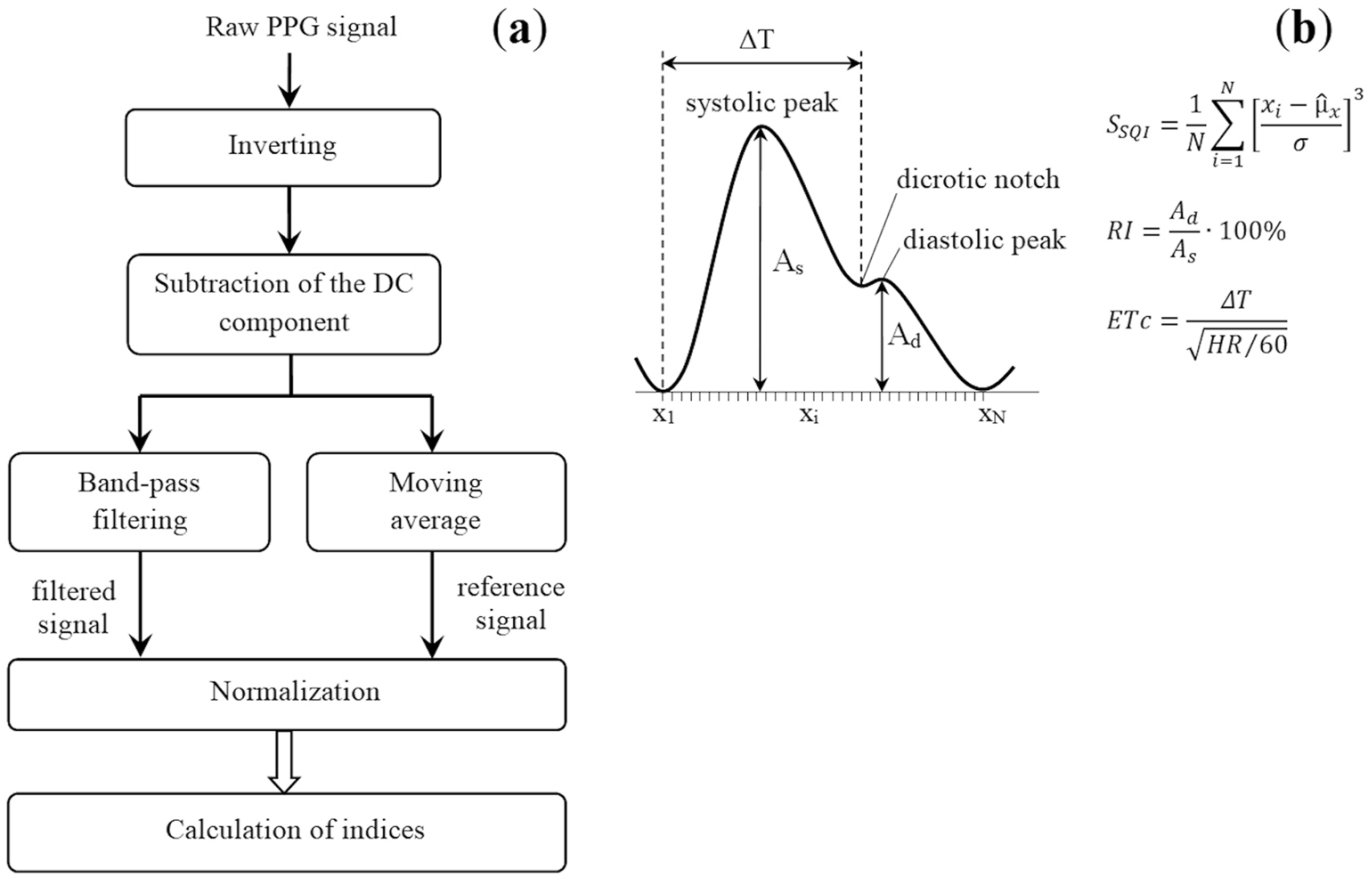
(**a**) Flowchart of the digital processing of the PPG signal; (**b**) explanation of calculated indices of the pulse waveform. *HR* denotes heart rate (beats per minute).

Recorded signals were processed in the LabView software environment (National Instruments, USA). The processing algorithm is shown in Fig. 10a. First, the raw PPG signal was inverted and the DC component was subtracted from it. Inverting is a standard procedure in PPG and is intended for a more convenient representation of the pulse wave signal^1^. The DC component was calculated using a moving average with a window width of 1 s (number of signal points *N* = 320):

6 $$DC=\frac{1}{N}\sum_{i=1}^{N}{y}_{i},$$

where *y*_*i*_ is the value of the *i*-th sample of a PPG signal. Then each signal was processed by a band-pass filter and a moving average filter in parallel to obtain a reference signal. The moving average window width was 3.125 ms (*N* = 10) and was selected in such a way as to closely follow the contour of the raw signal.

Next, the signals were normalized to the amplitude of the systolic wave (*A*_*s*_)^35^. This kind of normalization makes it possible to correctly compare pulse wave signals obtained by different types of filtering. Thus, the output pulse wave signal was in the range of 0–1 rel. units. The processed signals were compared to the original reference signal, which was not filtered, to evaluate filter-induced changes in the pulse wave shape.

First, the influence of the bandwidth of the Butterworth band-pass filter on the pulse waveform was estimated. For this, the same signal was filtered in the frequency ranges with varying upper cutoff frequency: 0.1–10 Hz, 0.1–5 Hz and 0.1–2 Hz. This makes it possible to simulate different values of the central frequency of the passband in (5) and investigate its effect on the filter time delay. The obtained signals were compared with each other and with the reference signal. Next, we evaluated the effect of different IIR filters (Butterworth, Bessel, Elliptic, Chebyshev I and II) of different orders (2nd, 4th and 6th) on the PPG waveform. Changing the filter order allows us to simulate different values of the quality factor in (5).

To analyze filter-induced changes in the pulse waveform, we calculated the skewness signal quality index (*S*_*SQI*_)^45^. This index was borrowed from probability theory and is a measure of the symmetry (or the lack of it) of a probability distribution. It has been shown that the *S*_*SQI*_ is the optimal index for assessing PPG signals compared to other indices such as perfusion, kurtosis, entropy, etc.^45^. The increased skewness of the PPG signals reveals a more detailed morphology of the pulse waveform. It is defined in the time domain of a PPG signal as (see Fig. 10b):

7 $${S}_{SQI}=\frac{1}{N}\sum_{i=1}^{N}{\left[\frac{{x}_{i}-{\widehat{\mu }}_{x}}{\sigma }\right]}^{3},$$

where ${\widehat{\mu }}_{x}$ and *σ* are the empirical estimate of the mean and standard deviation of *x*_*i*_, respectively, and *N* is the number of samples in the PPG signal.

When analyzing filters of different types, we additionally calculated the reflection index (*RI*, %) and ejection time compensated (*ETc*, ms) from pulse waves in subjects with a pronounced dicrotic notch (*n* = 10). Such signals belong to Classes 1 and 2 in accordance with Dawber's classification^46^. *RI* is defined as the ratio of the amplitude of the diastolic wave *A*_*d*_ to the systolic wave amplitude *A*_*s*_ (see Fig. 10b). This index characterizes the tone of small arteries (arterioles), which has been repeatedly shown in experiments with the use of vasoactive drugs^10,11^.

Ejection time compensated (*ETc*) is expressed as $ETc=\Delta T/\sqrt{HR/60}$ (ms), where Δ*T* is the time from onset of the systolic upstroke to the closure of the aortic valve (see Fig. 10b), *HR* denotes heart beats per minute. It was established that the systolic time parameter *ETc* by PPG directly corresponds to the left ventricular ejection time (LVET) by applanation tonometry^9^. Therefore, *ETc* is suitable for estimation of the left ventricle function. A prolonged *ETc* could indicate heart insufficiency with impaired cardiac output and aortic valve problems like stenosis. An abnormally short *ETc* could indicate hyperthyroidism, diastolic hypertension, and a small left ventricle^9^.

Thus, *ETc* and *RI* indices characterize central and peripheral vascular properties, respectively. The calculation of indices for each subject was carried out for the entire recording period and averaged over all impulses.

### Statistical analysis

Statistical analysis was performed using the IBM SPSS Statistics v25 software (IBM Corp., USA). Medians and quartiles were calculated for variables. The statistical significance of differences in the indices between filtered and reference signal was calculated using the Wilcoxon signed-rank test for paired samples. A level of *p* < 0.05 was considered statistically significant.

### Ethics approval and consent to participate

The study protocol complies with the ethical principles of the Declaration of Helsinki (2013 revision) and was approved by the Independent Ethics Committee of the Moscow Regional Research and Clinical Institute (“MONIKI”) (Protocol No. 16 of 15 December 2022). Written informed consent was obtained from all participants.

## Data Availability

The data obtained and analysed during the current study are available from the corresponding author on reasonable request.

## Abbreviations

PPG: Photoplethysmography
IIR: Infinite impulse response
S_SQI_: Skewness signal quality index
RI: Reflection index
ETc: Ejection time compensated
FIR: Finite impulse response
LED: Light emitting diode
HR: Heart rate
LVET: Left ventricular ejection time
